# Magnetic Fe_3_O_4_@SiO_2_–Pt and Fe_3_O_4_@SiO_2_–Pt@SiO_2_ Structures for HDN of Indole

**DOI:** 10.3390/ma12233878

**Published:** 2019-11-24

**Authors:** Robinson Dinamarca, Verónica Valles, Brenda Ledesma, Cristian H. Campos, Gina Pecchi, Andrea Beltramone

**Affiliations:** 1 Facultad de Ciencias Químicas, Universidad de Concepción, Edmundo Larenas 129, 4030000 Casilla 160-C, Concepción, Chile; robidinamarca@udec.cl (R.D.); ccampos@udec.cl (C.H.C.); gpecchi@udec.cl (G.P.); 2 Facultad de Educación, Universidad de Concepción, Chile; 3 Centro de Investigación en Nanociencia y Nanotecnología (NANOTEC), Facultad Regional Córdoba, Universidad Tecnológica Nacional, Maestro López y Cruz Roja Argentina, 5016, Córdoba, Argentina; vvalles@frc.utn.edu.ar (V.V.); bledesma@frc.utn.edu.ar (B.L.); 4 Millenium Nuclei on Catalytic Processes towards Sustainable Chemistry (CSC), Pontificia Universidad Católica de Chile, Av Libertador Bernardo O’Higgins 340 (3542000) Santiago, Chile

**Keywords:** core@shell, platinum, mesoporous materials, indole HDN

## Abstract

The effect of a second porous SiO_2_ shell in the activity and selectivity of the Fe_3_O_4_@SiO_2_–Pt catalyst in the hydrodenitrogenation of indole is reported. The double Fe_3_O_4_@SiO_2_–Pt@SiO_2_ structure was prepared by coating Fe_3_O_4_ nanoparticles with tetraethyl orthosilicate (TEOS) with a further impregnation of 1.0 wt.% of Pt on the (3-aminopropyl)triethoxysilane functionalized Fe_3_O_4_@SiO_2_ structures. The second porous SiO_2_ shell, obtained by using a hexadecyltrimethylammonium bromide (CTAB) template, covered the Fe_3_O_4_@SiO_2_–Pt catalyst with a well-defined and narrow pore-sized distribution. The full characterization by TEM, inductively coupled plasma-optical emission spectroscopy (ICP-OES), XRD, and N_2_ adsorption isotherm at 77 K and vibrating sample magnetometry (VSM) of the catalysts indicates homogeneous core@shell structures with a controlled nano-size of metallic Pt. A significant effect of the double SiO_2_ shell in the catalytic performance was demonstrated by both a higher activity to eliminate the nitrogen atom of the indole molecule present in model liquid fuel and the improvement of the catalytic stability reaching four consecutive reaction cycles with only a slight conversion level decrease.

## 1. Introduction

The presence of polyaromatic and cyclic compounds in diesel negatively affects the environment and reduces the quality of diesel, as well as lowers the cetane number, which is indicative of the ease of ignition thereof. Hydrotreatment is one of the most efficient methods for the removal of N and S from refinery currents, as well as for the saturation of alkenes and aromatics [1,2,3,4]. It is important to highlight the multiplicity of reactions that come into play in hydrotreatment in this area of study. Taking into account the different elements that are considered contaminants in the refining processes of the petrochemical industry, within hydrotreatment we can distinguish hydrodesulfurization (HDS), hydrodenitrogenation (HDN), hydrodeoxygenation (HDO), hydrodemetalization (HDM), and hydrodearomatization (HDA). The HDS has been extensively studied because sulfur is a pollutant that is present in higher proportions in crude oils of lower quality and also as an important catalyst deactivator because of poisoning [5,6,7,8,9]. However, removal of nitrogen from organic compounds present in various fractions of crudes is harder than sulfur removal, which leads to the importance of the study of the HDN reaction. In this process of heterogeneous catalysis, the industry currently uses variations of the following combinations: Co(Ni)Mo(W)/Al_2_O_3_. In this arch for highly active and selective hydrotreatment catalysts for the oil industry, there are numerous works based on catalysts mainly with noble metals as active sites, since although they have a higher cost, the high conversions achieved justify their promising uses. Likewise, our previous works have shown that the acid support, the formation of bimetallic alloys, and the introduction of heteroatoms can greatly improve the performance of deposited metals in the active phase [10,11,12,13,14,15,16,17]. In this way of looking for more efficient catalysts, the supports with large surface areas have been greatly studied since they can achieve a great dispersion of the active phase, significantly improving the yield of the reactions. In this sense, the support materials have been the protagonists of various studies. Among the most studied are metal oxides, such as Al_2_O_3_ [4,5,6,7,8,9], and materials with different degrees of porosity, such as MCM (Mobil Composition of Matter), SBA, Santa Barbara Amorphous type material and zeolites [10,11,12,13,14,15,16,17,18].

Among the challenges that arise in the development of catalysts for the refining of crude oil, the factors associated with their reuse are decisive, including the characteristics that allow the separation of the catalyst from the reaction medium, as well as its recovery and the degree of activity that can be maintained in multiple reaction cycles. Accordingly, other interesting catalysts are core-shell nanoparticles (CSNs), designated as core@shell, that are formed with nuclei (inner materials) and shells (external material) at nanoscale. These types of materials have many applications, specifically in the field of heterogeneous catalysis, allowing the design, both in terms of the core and the casing, to achieve synergy between the two towards greater efficiency, yield, and selectivity. Depending on the constituent materials, they can be classified as CSN combinations of core shell: inorganic/inorganic, organic/organic, inorganic/organic, and inorganic/organic. Organic CSNs require polymerization techniques to prepare the organic core, shell, or both, while inorganic CSNs can be categorized as silica-based CSNs and metal-based CSNs. Silica-based CSNs have been extensively studied because silica is considered inert, that is, a simple dispersant of the active phase, although some authors have shown that they can react with metal precursors to form silicates of Ni, Co, Cu, Zn, and Ce with different reactivity [19,20,21,22,23,24,25,26,27,28,29]. Core@shell silica microspheres with ultra-small encapsulated nanoparticles of Pd have been highlighted as efficient and easily recyclable for the catalytic hydrogenation of various groups of olefins, alkynes, keto, and nitro groups [28,29]. One application of the combination of the properties of the core and the shell is the development of magnetically separable catalysts, a quality that represents a significant improvement when evaluating the reuse of a catalyst. Polshettiwar et al. [30] reported the synthesis of supported nanoparticles of Ni-ferrite with excellent magnetic recovery in hydrogenation and transfer hydrogenation reactions. Scähtz et al. [31] in their review article made an extensive analysis of various catalysts supported in ferrite nanoparticles, highlighting their properties for magnetic separation. The application of core@shell silica microspheres, with ultra-small encapsulated nanoparticles of Pt, in the catalytic hydrotreatment process was not found in the literature. The main objective of this work is to develop homogeneous core@shell structures with a controlled nano-size of metallic Pt and study the effect of the double SiO_2_ shell in the catalytic performance in the HDN of indole. 

## 2. Materials and Methods 

### 2.1. Synthesis 

All reagents were used without purification or treatment provided; these were acquired from Sigma^®^ (Darmstadt, Germany) and Merck® (Darmstadt, Germany) Company. The Fe_3_O_4_–NPs were synthesized by a solvothermal method following Long et al. [32], and FeCl_3_ (Merck^®^) was dissolved in a polyvinyl pyrrolidone (PVP K30, Sigma^®^) solution in ethylene glycol (EG, Merck^®^) with sodium acetate as nucleating agent. The mixture was transferred to a Teflon autoclave and isothermally treated at 200 °C for 8 h. The solid obtained was separated by magnetization and washed several times with absolute ethanol. The Fe_3_O_4_-core NPs were coated with SiO_2_ using the Stöber method [32]. Fe_3_O_4_–NPs were dispersed in a mixture of ethanol, water, and ammonia, and after that tetraethyl orthosilicate (TEOS, Merck^®^) was slowly added to the dispersion under stirring for 6 h. The Fe_3_O_4_@SiO_2_ solid was separated by magnetization and washed several times with an ethanol–water mixture. In order to promote an active Pt immobilization and dispersion on the surface of the material, the surface of the Fe_3_O_4_@SiO_2_ was functionalized with (3-aminopropyl)trimethoxysilane (AMPTS, Merck^®^, 1 mL g^−1^ of solid), a coupling agent refluxing in toluene for 24 h under mechanical stirring. The solid was separated by magnetization, washed with a toluene–acetone mixture, and dried in an oven at 50 °C for 12 h. The functionalized material was dispersed in a K_2_PtCl_6_ (Merck^®^) solution with an amount of precursor necessary to produce 1.0 wt.% systems with respect to Fe_3_O_4_@SiO_2_. The material was placed in contact with the solution for 3 h under mechanical stirring and then reduced with a fresh solution of NaBH_4_ (Merck^®^). The second coating of SiO_2_ was deposited on the Fe_3_O_4_@SiO_2_–Pt surface by a modified Stöber method reported previously [33] using TEOS as a precursor, triethanolamine (TEA) as a basic catalyst, and hexadecyltrimethylammonium bromide (CTAB, Sigma^®^) as a stabilized and soft template. Finally, the CTAB template was removed by ion exchange using ammonium nitrate in ethanol dissolution under reflux conditions, obtaining the Fe_3_O_4_@SiO_2_–Pt@mSiO_2_ material.

### 2.2. Characterization

The morphology and microanalysis of the structures were studied by scanning transmission electron microscopy with energy dispersive X-ray spectroscopy (STEM-EDS) using an FEI Tecnai ST F20 microscope (FEI, Hillsboro, OR, USA) operating at 200 kV. Up to 300 individual metal particles were counted for each catalyst, and the surface area-weighted mean Pt diameter (d_p_) was calculated using the software ImageJ 1.48 (Wayne Rasband, National Institute of Health, Bethesda, MD, USA). Adsorption isotherms were obtained at 77 K in a Micromeritics ASAP 2010 instrument (Norcross, GA, USA). X-ray powder diffraction profiles were obtained in a Rigaku Diffractometer with Cu K_α_ radiation (λ = 1.5418 Å) and a nickel filter (Rigaku, Tokyo, Japan). The magnetic behavior was studied using a Lakeshore series 7400 vibrating sample magnetometer (VSM) in an applied field of 20 kOe at 27 °C (Lakeshore, New Orleans, LA, USA). Pt and Fe contents were determined by inductively coupled plasma-optical emission spectroscopy (ICP-OES) with a Perkin–Elmer Optima 2100 DV instrument (Perkin Elmer, Waltham, MA, USA). The catalyst contents were determined after digestion in a 1:3 mixture of HNO_3_:HCl. X-ray source spectrophotometry (XPS) was obtained on a STAIB Instruments brand RQ-300 X-ray Source spectrophotometer (XPS RQ300/2, StaibInstrumente GmbH, Langenbach, Germany). The monochromatic radiation used as an excitation source is that of Al Kα (hν = 11486.6 eV) operated at 75 W.

### 2.3. Catalytic Activity

The hydrodenitrogenation reactions of indole were carried out at 250 °C and 15 atm of H_2_ and 500 rpm in a 600 mL stirred autoclave (Parr Pressure Reactor 4536, Parr Instrument Company, Moline, IL, USA). The typical procedure was as follows: 150 ppm of N as indole was dissolved in 50 mL of dodecane (0.01 mol L^−1^). The mixture was placed into the autoclave and the catalyst (250 mg) was transferred to the reactor. The reaction time was 8 h; samples were taken every hour. The products were analyzed with a HP 5890 Series II GC and HP-5 capillary column and identified by GC/MS.

## 3. Results and Discussion

### 3.1. Characterization

**Figure 1:**
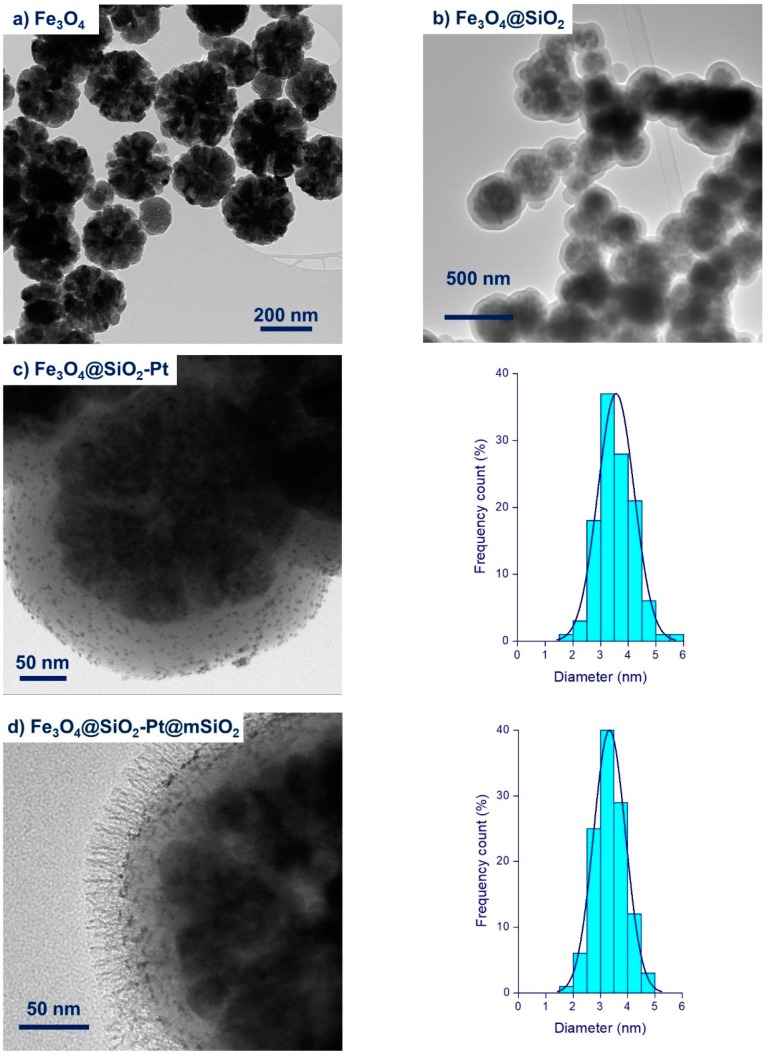
**HR-TEM** of the synthesized Fe_3_O_4_–NPs and core@shell structures. (**a**) Fe_3_O_4_; (**b**) Fe_3_O_4_@SiO_2_; (**c**) Fe_3_O_4_@SiO_2_-Pt; (**d**) Fe_3_O_4_@SiO_2_-Pt@mSiO_2_.

Figure 1 presents the high-resolution transmission electron microscopy (HR-TEM) for the image sequence obtained at each step of the catalyst synthesis; the Fe_3_O_4_-core image is shown in Figure 1a. Figure 1b reveals that the CSNs, where the Fe_3_O_4_ core is covered by a layer of SiO_2_, were close to 45 nm thick (Table 1). In Figure 1c,d, a successful impregnation-reduction process obtaining Fe_3_O_4_@SiO_2_–Pt and Fe_3_O_4_@SiO_2_–Pt@mSiO_2_ catalysts can be seen, respectively. Both catalysts present similar Pt size distribution and an average particle size of 3.5 nm (Table 1). The SiO_2_ coating generated by the Stöber method did not modify the distribution or increase the average size of the Pt–NPs on the surface of the material. The micrographs show the formation of a uniform coating on the surface of both catalysts, with channels perpendicular to the surface that correspond to the porosity of the material formed during the removal of the organic CTAB template used as a porosity directing agent. The preferential alignment of the surfactant and the silica oligomer with the core@shell in the performed structures is noticeable.

The catalysts were characterized by ICP-OES to determine the Pt content, as summarized in Table 1. The Fe_3_O_4_@SiO_2_–Pt catalyst contains slightly lower Pt than the nominal value, while for the Fe_3_O_4_@SiO_2_–Pt@mSiO_2_ catalysts the Pt loading is largely lower than the nominal. The larger difference in the Pt content for Fe_3_O_4_@SiO_2_–Pt@mSiO_2_ is attributed to the second mSiO_2_ shell of core@shell particles and not due to the leaching effect. When the mesoporous SiO_2_ shell is coated around the Fe_3_O_4_@SiO_2_–Pt structures, a uniform increase in the size of the second shell is observed, reaching 51 nm of thickness in mean diameter (see Table 1). The increase of the thickness provides a dilution of the Pt active phase compared to the Fe_3_O_4_@SiO_2_–Pt structures, in the same way as the increase of the SiO_2_ shell thickness. 

**Figure 2:**
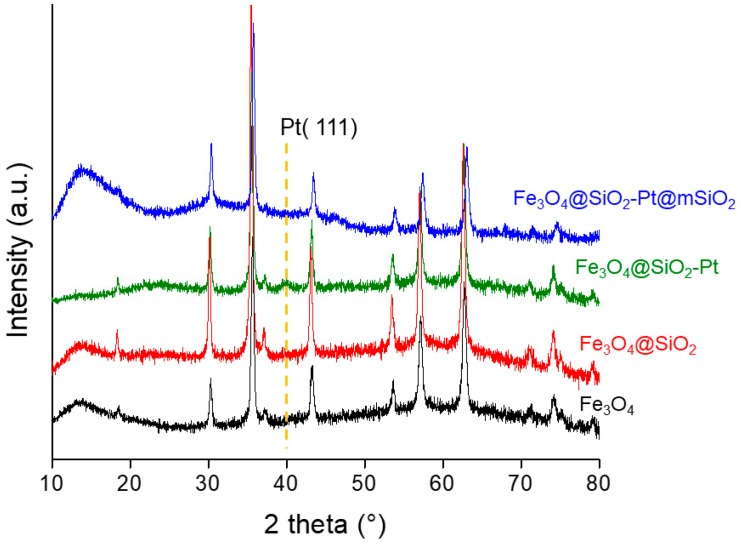
XRD of the synthesized Fe_3_O_4_–NPs and core@shell structures.

Figure 2 illustrates the XRD patterns of the synthesized materials. All systems show the diffraction peaks characteristic of magnetite, Fe_3_O_4_ (JCPDS 19-0629) [34]. The Fe_3_O_4_ phase was not modified during the SiO_2_ coating. This finding indicates that the crystalline phase of the material is restricted to the magnetite core. Only for the Fe_3_O_4_@SiO_2_–Pt is a diffraction peak at 2θ = 39° detected, corresponding to the surface metallic Pt (JCPDS 04–0802). The low intensity of this signal is attributed to the average particle size of the Pt clusters below the detection limit of the XRD technique. The high dispersion of the Pt crystalline phase is in line with the homogenous distribution of Pt–NPs on the surface of the SiO_2_ shell observed by HR-TEM, and the surface XPS technique confirms the presence of metallic Pt (see below).

**Figure 3:**
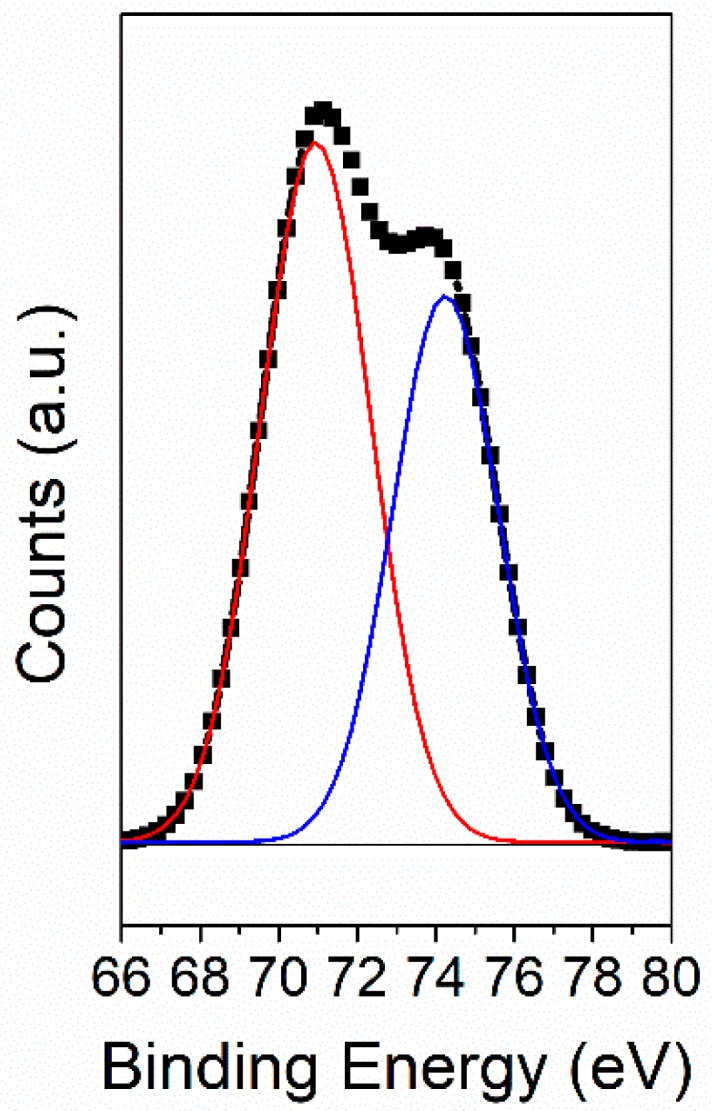
X-ray source spectrophotometry (XPS) of the Pt 4f_7/2_ of the Fe_3_O_4_@SiO_2_–Pt core@shell structure.

Figure 3 shows the spectra of Pt 4f_7/2_ for the Fe_3_O_4_@SiO_2_–Pt catalyst, with a BE of 70.9 eV attributed to the metallic Pt species. Surface metallic Pt was not detected in the system with the second SiO_2_ coating; this was attributed to the coverage with the second shell. 

**Figure 4:**
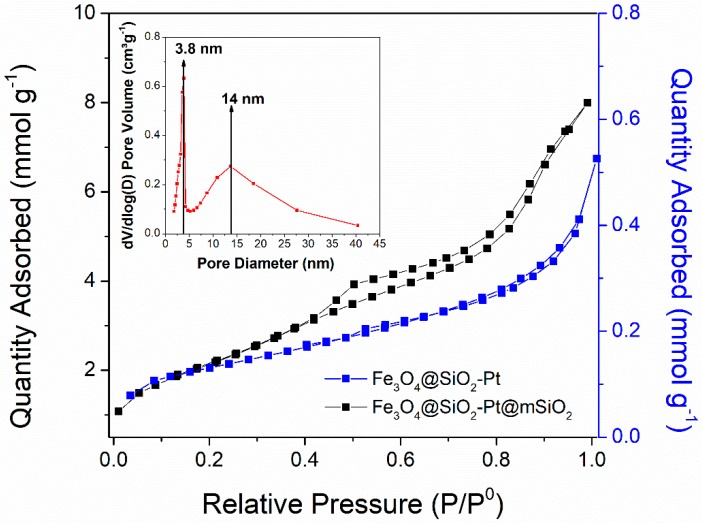
N_2_ adsorption isotherms and pore size distribution of the core@shell structures.

Table 1 presents the results obtained for S_BET_ calculated from the N_2_ adsorption-desorption isotherms at 77 K shown in Figure 4. The Fe_3_O_4_@SiO_2_–Pt shows an isotherm typical of type II materials and a S_BET_ value of 11 m^2^ g^−1^. After the deposition of the second shell, the isotherm changes to mesoporous type IV materials with a large increase in the specific area. The catalyst Fe_3_O_4_@SiO_2_–Pt@mSiO_2_ displayed an isotherm type IV with a hysteresis loop type H3 pores typical for cylindrical pores according to the IUPAC (International Union of Pure and Applied Chemistry) classification [35]. The isotherm exhibits two distinguished inflections in the adsorbed amount. The first one at P/P^0^ = 0.4–0.6 is attributed to the pores created after CTAB micelles removal [36,37], and the second one at P/P^0^ close to 0.8 which can be ascribed rather to the effect of interparticle voids present between some coalesced aggregates of the material [38]. The pore size distribution shows a bimodal distribution with a narrow distribution centered at 3.8 nm and a wider distribution centered at 14 nm associated with the interstitial spaces of the aggregates of the particles. The narrow distribution of mesopores at 3.8 nm is attributed to the removed CTAB organic template. These results are in line with both the shape of isotherm’s hysteresis loop and the TEM characterization for the observed core@shell aggregates. 

**Figure 5:**
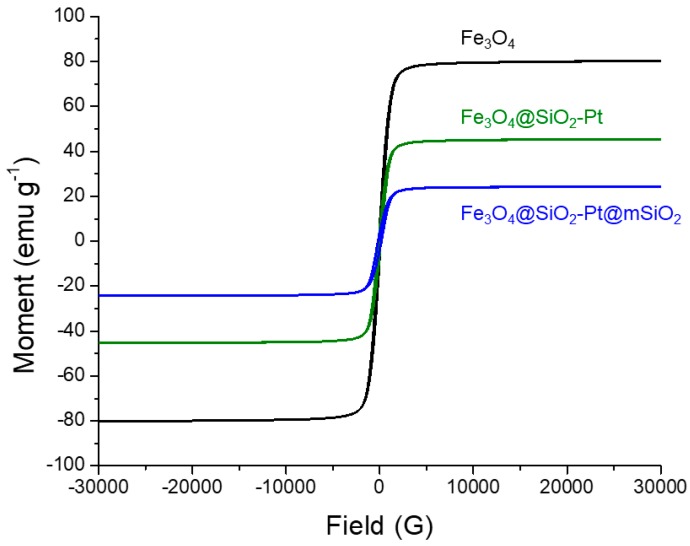
Vibrating sample magnetometer (VSM) of the synthesized Fe_3_O_4_–NPs and core@shell structures.

Fe_3_O_4_@SiO_2_–Pt and Fe_3_O_4_@SiO_2_–Pt@mSiO_2_ materials exhibit the ferromagnetic properties of the started Fe_3_O_4_–NPs, as can be seen in Figure 5. The magnetic saturation (M_s_) value of the Fe_3_O_4_–NPs changes with subsequent coverage, due to the relative decrease of Fe_3_O_4_ content with respect to the presence of the SiO_2_ shell. The Fe_3_O_4_ core corresponds to magnetization curves with an absence of a hysteresis cycle, characteristic of superparamagnetic materials. The decrease of the M_s_ of 91 emug^−1^ for magnetite phase (Fe_3_O_4_) to 45 emu g^−1^ for Fe_3_O_4_@SiO_2_–Pt and 25 emu g^−1^ for Fe_3_O_4_@SiO_2_–Pt@mSiO_2_ is a result of the effect of the coating with SiO_2_. Even though the relative lower range of value of M_s_ 25–45 emug^−1^ was enough for the efficient removal below 1 min of the micro-sized particles, it reflected the ability of these catalysts to respond to an external magnetic field, which allows for quick separation from the liquid phase [39].

### 3.2. Catalytic Activity

**Scheme 1:**
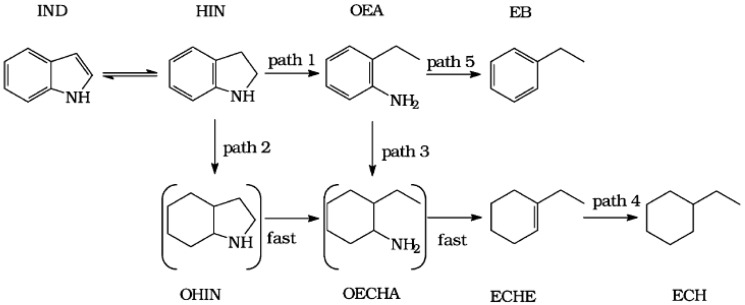
Reaction pathways for hydrodenitrogenation (HDN) of indole: indole (IND), indoline (HIN), o-ethylaniline (OEA), ethylbenzene (EB), octahydroindole (OHIN), o-ethylcyclohexylamine (OECHA), ethylcyclohexene (ECHE), and ethylcyclohexane (ECH).

The reaction network of the hydrodenitrogenation of indole proposed by Zhang and Ozkan is shown in Scheme 1 [40]. According to this pathway, ethylcyclohexane (ECH) and ethylbenzene (EB) are the two main products from HDN of indole. Path 1 indicates the hydrogenolysis of indoline (HIN) to o-ethylaniline (OEA) and path 2 indicates the hydrogenation of indoline (HIN) to octahydroindole (OHIN). A secondary route from o-ethylaniline (OEA) (path 3) to o-ethylcyclohexylamine (OECHA) and then to ethylcyclohexene (ECHE) is also included. The only identified products, after the experiments performed in this work, were indoline (HIN), o-ethylaniline (OEA), ethylbenzene (EB), and ethylcyclohexane (ECH); these accounted for almost 95% of the total products. 

**Figure 6:**
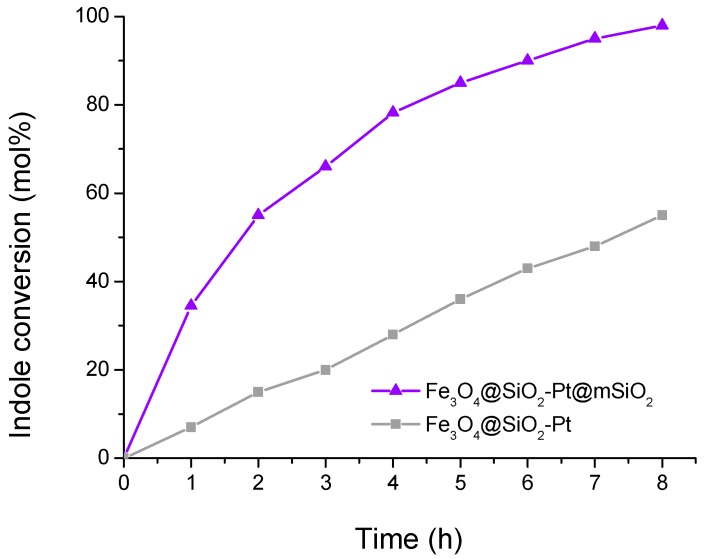
Indole conversion over the synthesized catalysts.

**Figure 7:**
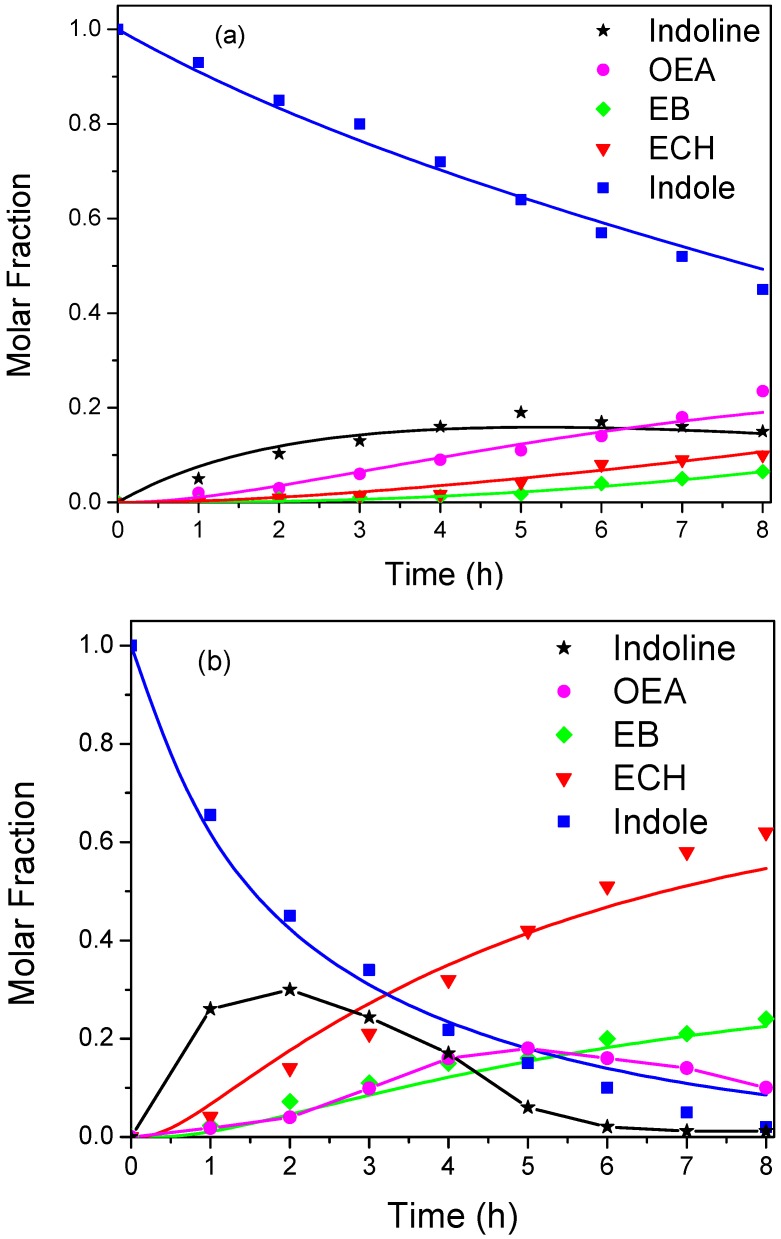
Molar fraction of indole and products in HDN reaction (**a**) Fe_3_O_4_@SiO_2_–Pt; (**b**) Fe_3_O_4_@SiO_2_–Pt@mSiO_2_.

Figure 6 shows the conversion of indole as a function of time for the two synthesized catalysts. It is clearly observed that the catalyst with the mesoporous silica coating Fe_3_O_4_@SiO_2_–Pt@mSiO_2_ is much more active than the uncoated catalyst, reaching a complete conversion at 8 h of reaction, while Fe_3_O_4_@SiO_2_–Pt only reaches 50% conversion. Figure 7 shows the molar fraction of the reaction system for both catalysts. In the case of Fe_3_O_4_@SiO_2_–Pt (Figure 7a), we can observe that indoline is formed first, then OEA, and that the appearance of EB and ECH is very slow. Contrary for Fe_3_O_4_@SiO_2_–Pt@mSiO_2_ (Figure 7b), the consumption of indole and the appearance of EB and especially ECH (the denitrogenated products) is very fast. The percentage of denitrogenated products (% HDN) was calculated and listed in Table 2. In the table, the results were compared with those of a typical HDN catalyst NiMo/Al_2_O_3_ Criterion DN200 [11]. This catalyst was presulfided according to our previously reported results [11]. Catalyst Fe_3_O_4_@SiO_2_–Pt@mSiO_2_ reached a higher % HDN value compared with the other two catalysts. A control reaction was carried out using Fe_3_O_4_@SiO_2_ as a catalyst under the same conditions and no activity was obtained, confirming the non-catalytic activity of the support.

The better activity of Fe_3_O_4_@SiO_2_–Pt@mSiO_2_ compared with the NiMo/Al_2_O_3_ catalyst can be related to the high hydrogenating capacity of platinum in Fe_3_O_4_@SiO_2_–Pt@mSiO_2_. In the case of both core@shell structures, it is very clear that the difference in activity is due to the presence of the second porous shell and not to the dispersion of the platinum nanoparticles. 

The large difference in activity has to be explained in relation to the presence of the mesoporous silica layer and in the greater surface area of Fe_3_O_4_@SiO_2_–Pt@mSiO_2_ compared with Fe_3_O_4_@SiO_2_–Pt. Fe_3_O_4_@SiO_2_–Pt@mSiO_2_ possesses cylindrical channels perpendicular to the surface that corresponds to the porosity of the material formed during the removal of the organic template. The synthesis of such coating was carried out using triethanolamine (TEA), which confers a basic property, leading to a negatively charged surface of the SiO_2_. Moreover, due to the removal of CTAB that was performed by ion exchange using ammonium nitrate in ethanol dissolution, this exchange by NH_4_^+^ cations enables adherence as counterions. The presence of these cations generates a slight surface acidity in the porous SiO_2_ of the second coating [41] which benefits HDN reactions, as we have reported previously [11]. This is also evidenced in the larger amount of ECH with respect to EB for the Fe_3_O_4_@SiO_2_–Pt@mSiO_2_ catalyst, similar to other acid catalysts [11]. On the other hand, in line with previous reports for heterogeneous core-shell catalysts with porous coatings, the porosity of the second coating generates a confinement effect into the cylindrical pores that favor the adsorption of the reactants and the re-adsorption of the intermediates of the reaction, increasing the contact time between intermediates and the platinum active catalytic species [42].

#### Reutilization Study

**Figure 8:**
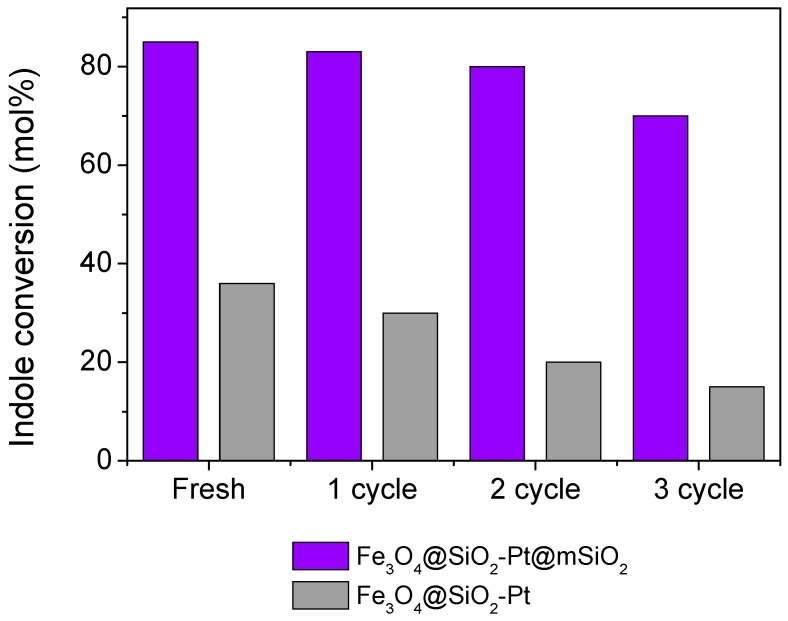
Reutilization study of the catalysts at 5 h of reaction time.

Catalyst reuse is important from an industrial point of view. In this case, the catalysts have been tested during four catalytic cycles. The samples were washed several times with a mixture of methanol and water previous to the reaction. Figure 8 shows that the activity slightly decreases after the third recycle in the case of Fe_3_O_4_@SiO_2_–Pt@mSiO_2_, but the loss in activity is higher in the case of Fe_3_O_4_@SiO_2_–Pt. The higher stability of Fe_3_O_4_@SiO_2_–Pt@mSiO_2_ could be due to the presence of the second porous silica coating that inhibits the loss of the metal charge and prevents sintering. The stabilization achieved after the second layer of silica allows the successive reuse of the catalyst. In addition, the magnetic characteristics of this catalyst facilitates the separation process, which is very important from the practical and economic perspective in any industrial process.

## 4. Conclusions

In this work, we studied the effect of a second porous SiO_2_ shell in the activity and selectivity of the Fe_3_O_4_@SiO_2_–Pt catalyst in the hydrodenitrogenation of indole. The core–shell structures were applied for the first time in the indole HDN process. We found that the presence of a mesoporous SiO_2_ coating dramatically increases the activity of the catalyst. The great difference in activity was explained in terms of a confinement effect of the intermediate’s products of the reaction in the second SiO_2_ layer. This coating of slightly acidic character, and with parallel mesochannels, favored the re-adsorption and transformation into denitrogenated products, allowing the Fe_3_O_4_@SiO_2_–Pt@mSiO_2_ catalyst to remain active after several catalytic cycles, and its magnetic character allows its easy separation and recovery.

## Figures and Tables

**Table 1:** HR-TEM characterization, inductively coupled plasma-optical emission spectroscopy (ICP-OES), S_BET_, and Pt 4f_7/2_ binding energy.

Catalysts	Core Mean Diameter (nm)	Thickness SiO_2_ Mean Diameter (nm)	Pt Mean Diameter (nm)	Pt Content (%)	S_BET_ (m^2^ g^−1^)	Pt 4f_7/2_ (eV)
Fe_3_O_4_@SiO_2_–Pt	229 ± 75	45 ± 12	3.6 ± 1.0	0.7 (1.0) ^(2)^	11	70.9
Fe_3_O_4_@SiO_2_–Pt@mSiO_2_	231 ± 42	96 ± 15 (51 ± 14) ^(1)^	3.4 ± 0.8	0.35 (1.0) ^(2)^	178	--

^(1)^ Second shell thickness SiO_2_ mean diameter in brackets; ^(2)^ nominal value in brackets.

**Table 2:** Indole conversion and % HDN of the catalysts at reaction time of 8 h.

Catalysts	Indole Conversion	% HDN ^1^
Fe_3_O_4_@SiO_2_–Pt	55	30
Fe_3_O_4_@SiO_2_–Pt@mSiO_2_	98	88
NiMo/Al_2_O_3_ [11]	50	30

**^1^**% HDN= 100 × (ECH + EB)/(ECH+EB+OEA+HIN).
